# Diagnostic performance and inter-observer concordance in lesion detection with the automated breast volume scanner (ABVS)

**DOI:** 10.1186/1471-2342-13-36

**Published:** 2013-11-12

**Authors:** Sebastian Wojcinski, Samuel Gyapong, André Farrokh, Philipp Soergel, Peter Hillemanns, Friedrich Degenhardt

**Affiliations:** 1 Department of OB/GYN, Hannover Medical School, OE 6410, Carl-Neuberg-Straße 1, 30625 Hannover, Germany; 2 Department of OB/GYN, Franziskus Hospital, Bielefeld, Germany

**Keywords:** Breast cancer, Automated breast ultrasound, Automated breast volume scanner, ABVS, Inter-observer concordance, Screening

## Abstract

**Background:**

Automated whole breast ultrasound scanners of the latest generation have reached a level of comfortable application and high quality volume acquisition. Nevertheless, there is a lack of data concerning this technology. We investigated the diagnostic performance and inter-observer concordance of the Automated Breast Volume Scanner (ABVS) ACUSON S2000™ and questioned its implications in breast cancer diagnostics.

**Methods:**

We collected 100 volume data sets and created a database containing 52 scans with no detectable lesions in conventional ultrasound (BI-RADS®-US 1), 30 scans with benign lesions (BI-RADS®-US 2) and 18 scans with breast cancer (BI-RADS®-US 5).

Two independent examiners evaluated the ABVS data on a separate workstation without any prior knowledge of the patients’ histories.

**Results:**

The inter-rater reliability reached fair agreement (κ=0.36; 95% confidence interval (CI): 0.19-0.53). With respect to the true category, the conditional inter-rater validity coefficient was κ=0.18 (95% CI: 0.00-0.26) for the benign cases and κ=0.80 (95% CI: 0.61-1.00) for the malignant cases.

Combining the assessments of examiner 1 and examiner 2, the diagnostic accuracy (AC), sensitivity (SE) and specificity (SP) for the experimental ABVS were AC = 79.0% (95% CI: 67.3-86.1), SE = 83.3% (95% CI: 57.7-95.6) and SP = 78.1% (% CI: 67.3-86.1), respectively.

However, after the ABVS examination, there were a high number of requests for second-look ultrasounds in up to 48.8% of the healthy women due to assumed suspicious findings in the volume data.

In an exploratory analysis, we estimated that an ABVS examination in addition to mammography alone could detect a relevant number of previously occult breast cancers (about 1 cancer in 300 screened and otherwise healthy women).

**Conclusions:**

The ABVS is a reliable imaging method for the evaluation of the breast with high sensitivity and a fair inter-observer concordance. However, we have to overcome the problem of the high number of false-positive results. Therefore, further prospective studies in larger collectives are necessary to define standard procedures in image acquisition and interpretation. Nevertheless, we consider the ABVS as being suitable for integration into breast diagnostics as a beneficial and reliable imaging method.

## Background

Today, we can expect that more than one million women will be newly diagnosed with breast cancer each year [1]. The most recent data were reported in 2000, when about 400,000 women died from breast cancer, which represented 1.6 percent of all female deaths [2]. Nations with the highest cancer rates include the U.S.A, Italy, Australia, Germany, the Netherlands, Canada and France [3]. Secondary prevention, i.e. the early detection of usually curable stages of breast cancer, has moved into the very focus of all healthcare systems. While the incidence of breast cancer remains considerably high, we notice a relevant decline in cancer mortality in numerous countries. This effort can partly be explained by new and innovative therapies, but there is sound evidence that advances in early detection probably play the most decisive role [4]. Hence, the early detection of breast cancer has moved into the central focus of primary healthcare. This success could only be achieved because of improvements in imaging technologies and a higher degree of health awareness.

Breast cancer screening programs are based on radiologic examinations of the breast. Additional imaging modalities are only indicated if suspicious or unclear findings are present in the mammogram. Mammography has demonstrated excellent sensitivity, specificity and inter-observer concordance [5,6]. Nevertheless, the diagnostic accuracy of breast ultrasound and magnetic resonance imaging (MRI) is as good as mammography. However, these two modalities imply a number of major disadvantages. Breast MRI is an expensive and complex technology. Moreover, MRI has insufficient specificity and, consequently, produces a relevant number of false-positive findings. Ultrasound, on the other hand, is observer-dependent, time-consuming and the examiner has to be present at the time of image acquisition. Furthermore, only subjectively chosen screenshots from the ultrasound examination are printed and/or stored. Mammography has the advantage that the examination is rapid, standardizable and cost efficient. The generation of the mammogram can be performed by medical assistant personnel and the stored images allow second-readings and follow-ups. However, merely focusing on detection rates and diagnostic accuracy, breast ultrasound, breast MRI or even the combination of several imaging technologies may be superior to mammography alone.

So far, breast ultrasound is the most commonly accepted and reliable diagnostic method for women with clinically or radiologically suspicious breast lesions [7]. Furthermore, bilateral whole breast ultrasound has even demonstrated diagnostic advantages in screening asymptomatic women [8-12]. Nevertheless, after a comparison of the advantages and disadvantages of the various imaging techniques, mammography has become the most common, reliable and efficient screening method. This attitude in breast cancer screening may change someday, when technical advances allow further improvements of ultrasound and MRI. At this point, the concept of automated whole breast ultrasound emerges with the potential capability to overcome the inherent deficits of hand-held breast ultrasound. Automated breast scanners can be easily operated by medical assistants. The stored volume data allow comfortable and time-efficient evaluation at anytime by a medical professional. The performance of second-readings by additional examiners and follow-up evaluations are unproblematic. The concept of automated breast ultrasound dates back to the 1970s [13].

In the current report, we present data concerning an up-to-date technology in automated ultrasound, the Automated Breast Volume Scanner (ACUSON S2000™ ABVS; Siemens Medical Solutions, Inc., CA, USA). The ABVS reconstructs 3D data sets of the entire breast volume from automatically acquired B-mode images. These data can be stored and analyzed on a separate workstation.

We evaluated whether or not breast lesions, previously detected by means of conventional ultrasound, could also be detected and correctly classified by independent examiners who only used ABVS data. Furthermore, we analyzed the inter-observer concordance and performed a model calculation to scrutinize the potential implications of the ABVS in a screening setting.

## Materials and methods

### General design and patient database

Our study was carried out at the Franziskus Hospital Breast Cancer Center in Bielefeld, Germany, between March 2010 and July 2011. For the ultrasound examinations, we used the Siemens ACUSON S2000™ ultrasound system with the integrated ABVS (Siemens Medical Solutions, Inc., CA, USA). Patients are generally referred to our outpatient department on account of specific diagnostic queries, such as palpable breast lesions, breast pain, suspicious mammograms and intensified screening in low-risk and high-risk populations. Usually, patients receive a clinical examination, mammography and conventional breast ultrasound as the standard diagnostic methods, as well as subsequent examinations whenever necessary. Additionally, we offer an optional ABVS examination to all our patients within the routine practice of our breast cancer center. As a diagnostic standard, ultrasound pictures are categorized according to the Breast Imaging Reporting and Data System criteria of the American College of Radiology (ACR BI-RADS®-US) [14]. Study participants were recruited from this population. Patients with a final categorization of BI-RADS®-US 1, 2 or 5 in the conventional ultrasound examination were regarded as suitable for our study. BI-RADS®-US categories 0, 3 and 4 involve breast lesions of questionable dignity. As the focus of our study was on the detection of evidently benign or malignant lesions, we excluded patients with BI-RADS®-US 0, 3 and 4. Patients with a bra cup size greater than D, a history of breast surgery, inflammatory conditions of the breast, skin disorders or psychiatric disorders were also excluded. Patients who met the inclusion criteria and agreed to receive an additional ABVS examination entered our study. In this way, we performed 100 breast examinations with the ABVS and, consequently, we created a database containing 100 3D-scans with BI-RADS®-US 1, 2 or 5 findings in the volume.

All data were obtained using a standard of care clinical protocol and approved equipment. Therefore, the responsible ethics committee did not demand additional approval for this non-interventional case study. Although the requirements of individual informed consent were waived, we decided that all study participants signed an additional informed consent form before the ABVS examination.

Two independent examiners evaluated the cases from the anonymized ABVS database. We compared the performance of these two examiners (ABVS, experimental method) with each other and with the results from the conventional ultrasound (gold standard).

### Technical background of the ABVS ultrasound system

**Figure 1:**
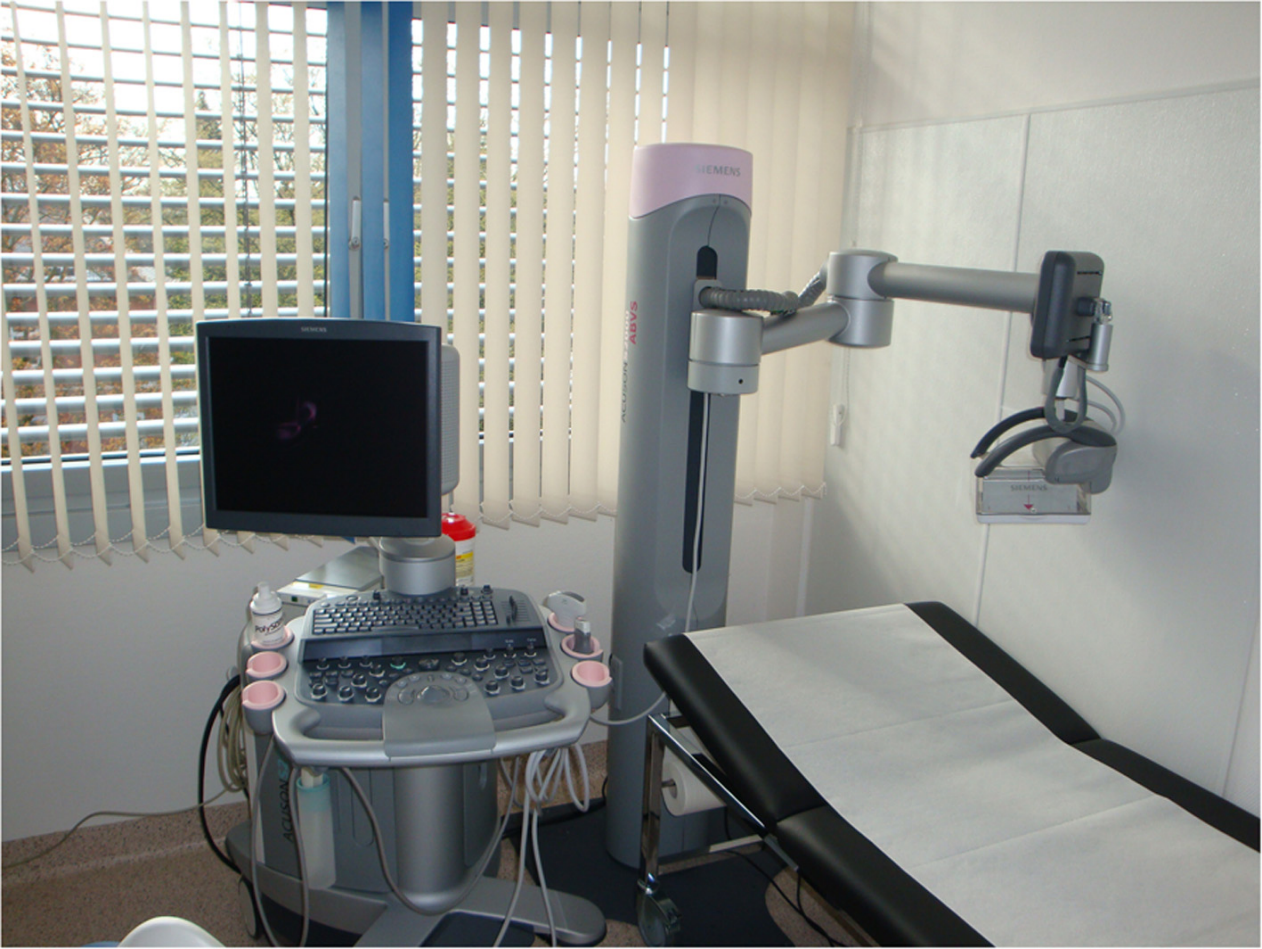
**ACUSON S2000™ ABVS.** On the left-hand side is the ACUSON S2000™ ultrasound machine, on the right-hand side is the 14L5BV volume transducer attached to a mechanical arm.

**Figure 2:**
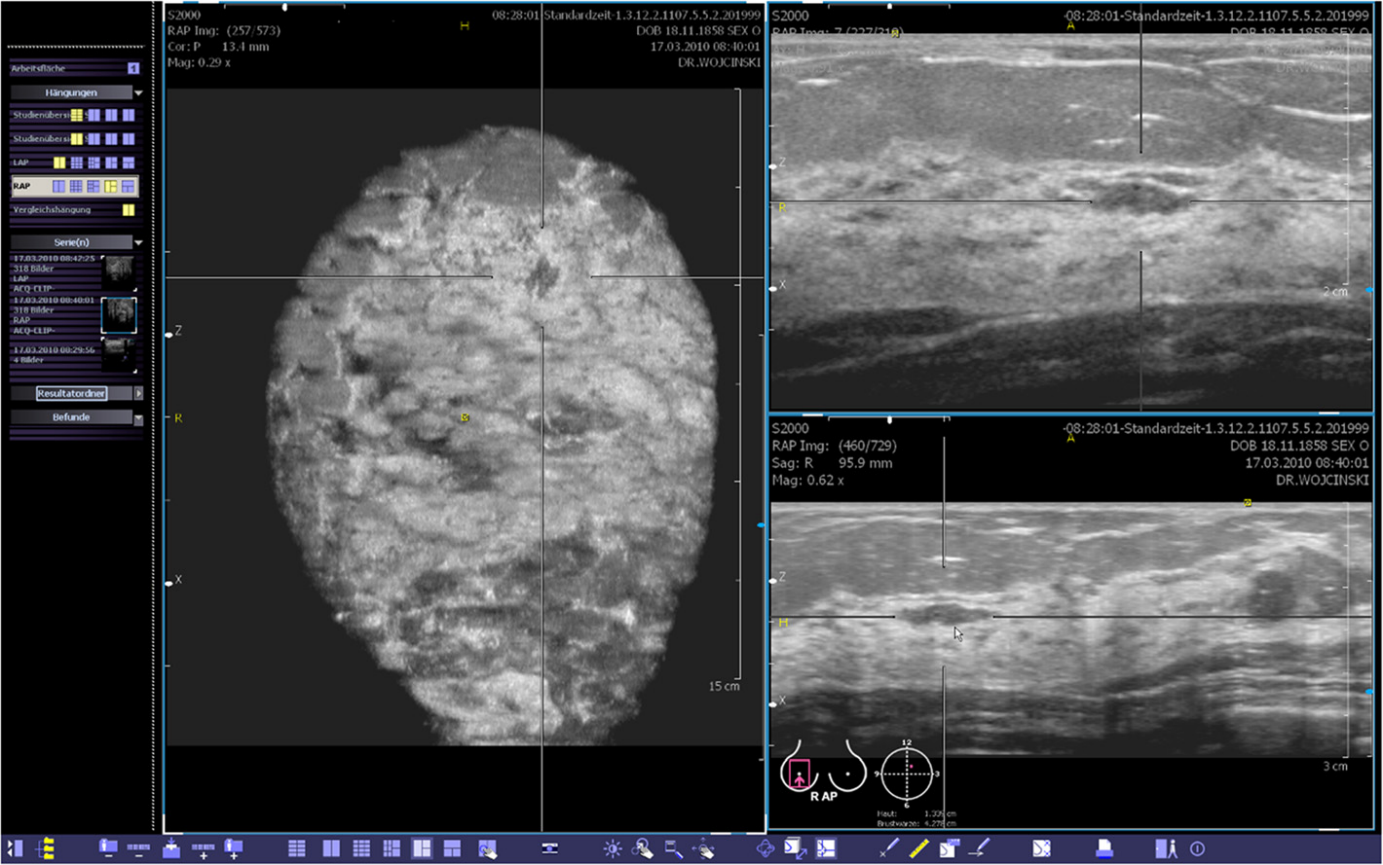
**ABVS data on the workstation.** This view provides the coronal (left), transverse (upper right) and sagittal (lower right) planes. The yellow spot marks the position of the nipple. The body marker indicates that this volume was acquired at the apex of the right breast. A plane of interest can be selected and marked by two orthogonal lines. Then, the corresponding cross-sections are calculated in real-time. Finally, the images can be optimized by adjusting magnification, brightness and contrast.

For our study we used the ACUSON S2000™ ABVS (Siemens Medical Solutions, Inc., CA, USA), an ultrasound system that automatically acquires full-field volumes of the breast (Figure 1). The system is equipped with an ultra-wide linear transducer (Siemens 14L5BV, 14 MHz, 15.4 cm, and 768 piezoelectric elements). While automatically sweeping over the breast, this ultrasound probe covers a distance of 16.8 cm in approximately one minute, acquiring about 300 high-resolution slices for post-processing (resolution: axial = 0.09 mm, lateral = 0.16 mm, sagittal = 0.44 mm). These images are the source for the creation of the 3D data sets. A separate workstation provides comprehensive tools for image analysis and manipulation of the volume data (Figure 2). The secondary images are calculated from the acquisition volume in real-time. Details concerning the ABVS have been described elsewhere [15,16].

### Conventional B-mode ultrasound examinations (gold standard)

Conventional B-mode ultrasound examinations were performed by the author SW, a DEGUM (Deutsche Gesellschaft für Ultraschall in der Medizin, German society for ultrasound in medicine) level II certified senior consultant in gynecology with 8 years’ experience in breast ultrasound [17]. This examiner also knew the results of the other imaging modalities when available (mammography, magnetic resonance imaging) and, therefore, defined the reference standard for the interpretation of the volume data sets. The conventional ultrasound was carried out with the ACUSON S2000™ using a standard linear transducer (Siemens 18 L6 HD, 5.5-18 MHz, 5.6 cm).

After having completed these diagnostic steps, eligible patients were additionally examined using the ABVS.

According to the national regulatory authority statutes, breast US systems have to fulfill basic technical requirements and undergo regular quality control measures [18]. These standards applied to the equipment used in our study.

### ABVS examinations (experimental method)

**Figure 3:**
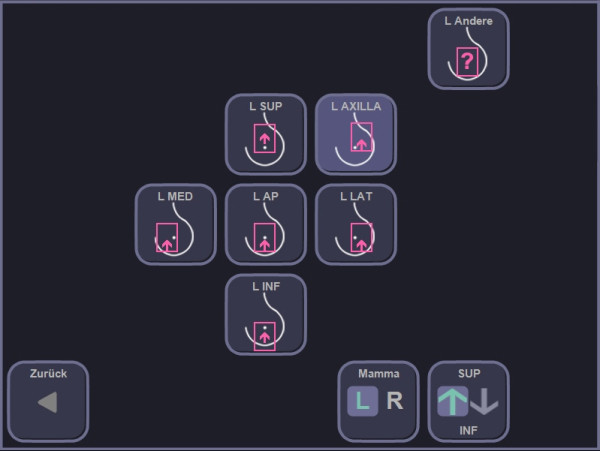
Predefined positions of the scanner for the left breast that are used to cover the entire volume.

The author SW performed the experimental ABVS examination in order to create 3D data sets. For the ABVS examination, the patients were in the supine position with the ipsilateral hand placed on the head. Depending on the size of the breast, the examiner chose the number of scans to be taken from each side. Usually, breasts with a bra cup size A or B can be fully displayed by performing two volume scans (medial and lateral, Figure 3). In larger breasts, it is frequently necessary to choose additional views (usually a separate view of the apex and the axillary process of the breast).

In order to guarantee sufficient contact with the skin, a replaceable membrane was attached to the transducer surface according to the manufacturer’s instructions. Next, the transducer was positioned on the breast with slight pressure. An automated scan took between 55 and 65 seconds. Finally, the entire set of volume scans was sent to the workstation.

### Interpretation of the experimental ABVS data by two independent examiners

The authors SG (examiner 1) and AF (examiner 2) performed the independent interpretation of the ABVS data sets. Both, SG and AF, are DEGUM level I certified senior residents in gynecology with 5 years’ experience in breast ultrasound [17].

The independent examiners exclusively analyzed the volume data sets without any knowledge of the patients’ histories, clinical findings or results of other imaging modalities. Furthermore, they had no information about the proportion of BI-RADS®-US 1, 2 and 5 cases in the database.

The following standard procedure was applied to the systematic analysis of the ABVS data: Initially, the entire volume was explored in the coronal plane moving slowly (i.e. in thin slices) from the skin to the chest wall. During this process, the examiner marked all mass lesions with the system’s default tool. Next, the examiner evaluated all of the selected lesions by displaying them in the sagittal and axial planes. Finally, the examiner assigned the lesions a category according to the ACR BI-RADS®-US system. Overall interpretation times usually range from 4 to 10 min per case.

Despite the fact that the examiner knew that there were no BI-RADS®-US 0, 3 or 4 cases in the database, he was allowed to categorize lesions as BI-RADS®-US 0, 3 or 4 whenever he requested a second-look ultrasound in order to further assess uncertain lesions.

When a second-look ultrasound was requested for a lesion that eventually turned out to be benign, the result of the AVBS examination was defined as “non-concordant” (false-positive). On the other hand, when a second-look ultrasound was requested for a lesion that turned out to be malignant, the result was classified as “concordant” (true-positive) because the cancer could then be correctly detected in the subsequent conventional ultrasound (Table 1).

**Table 1:** Methods

			**Evaluation of ABVS data (experimental method)**					
			**BI-RADS® ABVS**					
			**0**	**1**	**2**	**3**	**4**	**5**
**Evaluation in conventional ultrasound (gold standard)**	**BI-RADS®-US**	**0**	n.a.	n.a.	n.a.	n.a.	n.a.	n.a.
		**1**	FP	TN	TN	FP	FP	FP
		**2**	FP	TN	TN	FP	FP	FP
		**3**	n.a.	n.a.	n.a.	n.a.	n.a.	n.a.
		**4**	n.a.	n.a.	n.a.	n.a.	n.a.	n.a.
		**5**	TP	FN	FN	TP	TP	TP

Concerning the performance of the ABVS examination the above scheme was applied to define cases as true-positive (TP), true-negative (TN), false-positive (FP) or false-negative (FN).

### Statistical analysis

Microsoft® Office Excel® 2007 (Microsoft Corporation) was used for data collection.

Statistical analysis was performed by the author SW using MedCalc® 7.6 statistical software (MedCalc Software bvba, Belgium) and validated by the other authors.

In order to assess the diagnostic performance of the ABVS, we calculated sensitivity, specificity and accuracy for both examiners and used the Z-Test to compare the performance of examiner 1 with examiner 2. As our study population did not reflect the real prevalence of breast cancer, the positive and negative predictive values were estimated based on the Bayesian theorem using the reported prevalence of malignancies in screening collectives [19]. For the calculation of the 95% confidence levels, we used the Newcombe intervals with continuity correction [20].

The statistical analysis of the extent of agreement between the two raters was based on Cohen’s Kappa test. For the interpretation of κ-values we used the magnitude guidelines published by Landis and Koch, who characterized the values of κ<0 as indicating no agreement, κ 0–0.20 slight, κ 0.21-0.40 fair, κ 0.41-0.60 moderate, κ 0.61-0.80 substantial, and κ 0.81-1 as almost perfect agreement [21].

Furthermore, we assessed the correlation between the expected and the observed rate of second-look ultrasounds using the Chi-square test.

Statistical significance was assumed as p < 0.05 for all tests.

## Results

In our study population, the age ranged from 19 to 86 years (median 52 years). According to the BI-RADS®-US categorization, 52% (n = 52) of our cases were assigned as BI-RADS®-US 1, 30% (n = 30) had BI-RADS®-US 2 lesions and 18% (n = 18) of the cases had BI-RADS®-US 5 lesions. All BI-RADS®-US 5 lesions were confirmed with histological specimens. The mean tumor size for malignant and benign lesions was 22.0 mm (range 13 to 55) and 16.7 mm (range 8 to 36), respectively.

### Inter-rater reliability

The concordance between examiner 1 and examiner 2 concerning the correct clinical decision of whether the patient should undergo a control ultrasound due to a suspicious finding or whether the patient should be defined as healthy as there is no suspicious lesion is shown in (Table 2). The inter-rater reliability reached fair agreement and the Cohen’s Kappa value was κ=0.36 (95% CI: 0.19-0.53).

**Table 2:** Concordance between examiner 1 and examiner 2 concerning the correct clinical decision of whether the patient should undergo a control ultrasound due to a suspicious finding in ABVS (due to BI-RADS® 0,3,4 or 5) or whether the patient should be defined as healthy as there is no suspicious lesion in ABVS (i.e. BI-RADS® 1 or 2)

		**Examiner 1 (SG)**		
		**BI-RADS®**	**BI-RADS®**	**Total**
		**ABVS**	**ABVS**	
		**1 or 2**	**0, 3, 4 or 5**	
		**(negative)**	**(positive)**	
**Examiner 2 (AF)**	**BI-RADS®**	34^a^	8	42
	**ABVS**			
	**1 or 2**			
	**(negative)**			
	**BI-RADS®**	25	33^a^	58
	**ABVS**			
	**0, 3, 4 or 5**			
	**(positive)**			
	**Total**	59	41	100

Distribution of cases by rater and by category. The inter-rater reliability coefficient calculates to κ = 0.36 (0.19-0.53).

^a^concordance between examiner 1 and examiner 2.

A more detailed breakdown of the concordance between examiner 1 and examiner 2 concerning the distinct BI-RADS® category in the ABVS examination is given in (Table 3). In this analysis, the inter-rater reliability also reached fair agreement and the Cohen’s Kappa value was κ = 0.27 (0.14-0.40).

**Table 3:** Concordance between examiner 1 and examiner 2 concerning the distinct BI-RADS® category in the ABVS examination

		**BI-RADS® ABVS**	**BI-RADS® ABVS**	**BI-RADS® ABVS**	**BI-RADS® ABVS**	**Total**
		**1**	**2**	**0, 3 or 4**	**5**	
		**(No finding)**	**(Benign finding)**	**(Unclear finding)**	**(Malignant finding)**	
**Examiner 2 (AF)**	**BI-RADS® ABVS**	20^a^	2	4	2	28
	**1**					
	**(No Finding)**					
	**BI-RADS® ABVS**	4	8^a^	2	0	14
	**2**					
	**(Benign finding)**					
	**BI-RADS® ABVS**	14	7	13^a^	9	43
	**0, 3 or 4**					
	**(Unclear finding)**					
	**BI-RADS® ABVS**	4	0	5	6^a^	15
	**5**					
	**(Malignant finding)**					
	**Total**	42	17	24	17	100

Distribution of cases by rater and by detailed BI-RADS® ABVS category. Concerning this detailed evaluation, the inter-rater reliability coefficient calculates to κ = 0.27 (0.14-0.40).

^a^concordance between examiner 1 and examiner 2.

### Inter-rater validity

With respect to the true category (benign cases and malignant cases), the inter-rater validity coefficient calculated to κ=0.31 (95% CI: 0.21-0.41). Focusing on the benign cases (n = 82), the conditional inter-rater validity coefficient was κ=0.18 (95% CI: 0.00-0.26), indicating slight agreement. Concerning the malignant cases, we found a Cohen’s Kappa value of κ=0.80 (95% CI: 0.61-1.00), indicating substantial to almost perfect agreement (Table 4).

**Table 4:** Distribution of cases by rater, reported and true categories

	**Examiner 1**		**Examiner 1**		
	**ABVS negative**		**ABVS positive**		
	**Examiner 2**	**Examiner 2**	**Examiner 2**	**Examiner 2**	**Total**
	**ABVS negative**	**ABVS positive**	**ABVS negative**	**ABVS positive**	
**Benign cases (conventional ultrasound negative)**	34^a^	22	8	18^a^	82
**Malignant cases (conventional ultrasound positive)**	0^a^	3	0	15^a^	18
**Total**	34	25	8	33	100

The inter-rater validity coefficient calculates to κ = 0.31 (95% CI: 0.21-0.41). The conditional inter-rater validity coefficient is κ=0.18 (95% CI: 0.00-0.26) for the benign cases and κ=0.80 (95% CI: 0.61-1.00) for the malignant cases.

^a^concordance between examiner 1 and examiner 2.

### Diagnostic performance of the ABVS

The sensitivity for examiner 1 and examiner 2 in detecting malignant lesions with the ABVS was 83.3% (95% CI: 57.7-95.6) and 100% (95% CI: 78.1-100.0), respectively. The diagnostic accuracy of the method was 71.0% (95% CI: 60.9-79.4) and 60.0% (95% CI: 49.7-69.5), respectively. The differences between examiner 1 and examiner 2 were statistically not significant. Nevertheless, specificity revealed to be quite low at 68.3% (95% CI: 57.0-77.9) and 51.2% (40.0-62.3), respectively, as there was a relevant number of requests for second-look ultrasounds or further examinations in the group of healthy patients after the evaluation of the ABVS data. As previously described in the methods, these cases had to be classified as false-positives if there was no cancer (Table 1). Moreover, specificity was significantly different between examiner 1 and examiner 2 (p = 0.001). The detailed results are shown in (Table 5).

**Table 5:** Performance of examiner 1 and examiner 2 using the ABVS data to classify the breast either healthy (BI-RADS® 1or 2) or suspicious of malignancy (BI-RADS® 0, 3, 4, or 5)

	**Examiner 1 (SG)**	**Examiner 2 (AF)**	**p**
**Sensitivity**	83.3%	100%	n.s. (0.059)
	(57.7-95.6)	(78.1-100)	
**Specificity**	68.3%	51.2%	0.001
	(57.0-77.9)	(40.0-62.3)	
**Accuracy**	71.0%	60.0%	n.s. (0.102)
	(60.9-79.4)	(49.7-69.5)	

In order to investigate the effect of a second reading of ABVS data, we performed a tentative analysis and combined the evaluations of examiner 1 and examiner 2. With respect to the low specificity, the following rules for the combination of assessments were applied: If both examiners agreed that a scan was suspicious, the evaluation was considered “positive”. If both examiners agreed that a scan was unsuspicious or disagreed (and only one examiner regarded the scan as unsuspicious), the evaluation was considered “negative”. In this scenario, the accuracy increased to 79.0% (95% CI: 69.5-86.3), the specificity increased to 78.1% (95% CI: 67.3-86.1) and the sensitivity remained acceptably high at 83.3% (95% CI: 57.7-95.6) (Table 6).

**Table 6:** Estimation of the performance of the ABVS in a scenario, where second-reading of the data is performed

	**At least one examiners judges ABVS as normal (negative)**	**Both examiners concordantly suspicious ABVS (positive)**	**Total**
**Benign cases (conventional ultrasound negative)**	64^a^	18^b^	82
**Malignant cases (conventional ultrasound positive)**	3^b^	15^a^	18
**Total**	67	33	100

Sensitivity, specificity and accuracy calculate to 83.3%, 78.0% and 79.0%, respectively. For the evaluation of the second-reading the following rules were applied: If both examiners agreed that a scan was suspicious, the evaluation was considered “positive”. If both examiners agreed that a scan was unsuspicious or disagreed and only one examiner regarded the scan as unsuspicious, the evaluation was considered “negative”.

^a^“true” evaluation.

^b^“false” evaluation.

### Rate of second-look ultrasounds

We expected 18 requests for second-look ultrasounds after the ABVS examination as there were 18 BI-RADS®-US 5 lesions in our database of 100 cases. We did not expect requests for the other 82 cases (BI-RADS®-US 1 or 2 lesions). However, the observed rate of second-look ultrasounds was significantly high, totaling 41 for examiner 1 and 58 for examiner 2, respectively (p < 0.001).

Therefore, the rate of second-look ultrasounds in healthy women (i.e. BI-RADS®-US 1 and 2) was 31.7% for examiner 1 and 48.8% for examiner 2.

Regarding the BI-RADS®-US 1 and 2 cases separately, there was a request for a second-look ultrasound in 33.3% and 53.3% (examiner 1 and examiner 2) of the women with BI-RADS®-US 2 lesions and in 30.8% and 46.2% of the women with no breast lesions at all (i.e. BI-RADS®-US 1).

### Model calculation and exploratory analysis

Based on our findings, we performed a model calculation to estimate how additional ABVS examinations could affect the detection rate of cancer in screening collectives.

As described in the literature, the prevalence of occult cancer that can be detected by additional ultrasound in women who already underwent mammography and clinical examination can be estimated to be between 0.3% and 0.4% [8-12]. Therefore, conventional ultrasound can detect one cancer in about 250 otherwise healthy women.

Using these numbers, the positive predictive value in a screening collective would calculate to 1.49% and the negative predictive value to 99.91%, respectively. Therefore, the new false-negative rate in a diagnostic setting using the ABVS would be about 0.09% instead of 0.4% (Table 7).

**Table 7:** Model calculation

	**Interpretation of the ABVS data by 2 raters**
**Performance of the ABVS in our study collective (n = 100)**	
**Prevalence of disease**	18.00%
**SE**	83.33%
**SP**	78.05%
**Rate of second look ultrasounds in healthy women (=100%-SP)**	21.95%
**Estimated performance of the ABVS in a screening collective of asymptomatic women**^**1**^	
**Prevalence of disease**^**2**^	0.40%
**PPV**^**3**^	1.49%
**NPV**^**3**^	99.91%
**FNR**^**3,4**^	0.09%

Based on our data concerning the performance with two examiners (second-reading of the ABVS data sets), the effect in a screening setting was estimated. (SE = sensitivity; SP = specificity; PPV = positive predictive value; NPV = negative predictive value; FNR = false negative rate).

^1^Asymptomatic women are defined as women, who have a normal mammogram and exhibit no symptoms

^2^As described in the literature, the prevalence of occult carcinomas that can be detected by conventional breast ultrasound can be estimated to be about 0.4%. This value resembles the theoretical false negative rate (FNR) of clinical examination and mammography alone.

^3^The new performance values of the ABVS are based upon the estimated prevalence of 0.4% in a screening collective.

^4^The new false negative rate is based upon a combination of clinical examination, mammography and ABVS in a screening collective.

**Figure 4:**
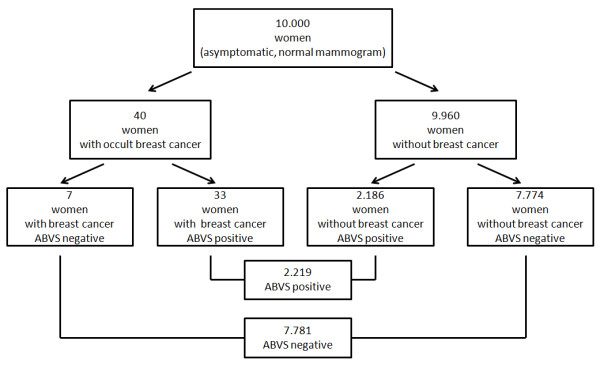
**Model calculation.** Derived from our results, we estimated the effect of an additional ABVS examination in 10.000 otherwise healthy women.

If 10,000 women were additionally screened with the ABVS, we could expect 2,219 positive scans resulting in the same number of second-look-ultrasounds, finally leading to 33 detected cancers. Therefore, one cancer would be detected in 67 second-look ultrasounds. 7,781 scans would be negative, and only seven cancers would be missed in this group (Figure 4).

## Discussion

### Primum non nocere

Innovative features for existing medical imaging modalities and even entirely new imaging technologies are constantly being developed [22,23]. Boosted by the industry, these technical novelties are rapidly made available for inpatient and outpatient care. Nevertheless, the new technologies often lack standardized imaging methodology evaluation and validation studies [24]. Therefore, the distinct indications for these methods, their definite diagnostic spectrum and, even more, their potential risk for the patient remain unclear to a certain degree. A universal principle for medical practice is “primum non nocere!” translated as “first, do no harm!” [25]. We bear this in mind when we address medical malpractice related to therapeutic interventions. However, malpractice can also occur in diagnostic procedures. The wrong interpretation of diagnostic findings, deficits in image acquisition, and, in particular, the wrong indication for a potentially beneficial examination may harm the patient in terms of unnecessary biopsies or overlooked breast cancer. Finally, the attending physician is charged with applying any new method with discernment. A prerequisite for this responsible utilization of new technologies is a profound knowledge of the powers and limitations of the same. Therefore, we encourage the reader to regard any new imaging technology critically until evident data has demonstrated a benefit for the patient beyond all doubt. Concerning the ABVS, such definite data is missing in the literature, but we regard our own results as a first approach to approve the ABVS for routine diagnostics.

### Performance of the ABVS in our study and in the literature

The focus of our current study was on the inter-rater agreement concerning the evaluation of ABVS data. We were able to demonstrate fair agreement for both the classification into the categories “positive/negative” (κ=0.36) and the detailed classification according to the BI-RADS® system (κ=0.27). Nevertheless, this performance could be improved. The conditional inter-rater validity coefficients for benign and malignant cases revealed that the concordance is almost perfect for the malignant cases (κ=0.80), but only slight for the benign cases (κ=0.18). These results correlate with the experience, that both examiners reached high sensitivity (83.3% and nominally 100%, respectively), but lower specificity (68.3% and 51.2%). In other words, the detection of malignant lesions can be precisely performed with the ABVS. Cancers are rarely missed with this technology. The examiners agree in those cases in which a suspicious lesion is present in the volume data. On the other hand, the examiners tend to suspect doubtful lesions even in the normal breast tissue of healthy women. Consequently, the ABVS has a low specificity and a high false-positive detection rate. This condition impairs the conditional inter-rater validity for the benign cases and leads to a high number of unnecessary second-look ultrasounds. In fact, when the ABVS system is used, we lose the ability to immediately further explore a questionable lesion by modifying factors such as compression, the orientation of the probe and the machine’s setting whilst acquiring the image in real-time. Ultrasound techniques such Doppler imaging or sonoelastography cannot be used either. Because the examiners cannot rely on standardized interpretation criteria or rules how to handle technical artifacts in the volume data, they seem to prefer false positive evaluation rather than missing a malignant lesion. Nevertheless, with accumulating data, growing experience and better diagnostic criteria this problem may be solved.

The consequence of a false positive result is the performance of an unnecessary second-look ultrasound, which is expensive, time-consuming and frustrating for both the patient and the medical professional. However, this second-look ultrasound implies no direct harm for the patient. A false-negative result may have a more serious implication as the diagnosis of malignancy is delayed, with a potentially worse clinical outcome for the patient.

In conclusion, as the false-negative rate is low we assume high patient safety and encourage the clinical use of the ABVS for breast diagnostics.

There is only limited data in the literature describing the inter-observer concordance in lesion detection with the ABVS:

In 2011, Shin et al. reported on 55 women with 145 breast masses who were examined with handheld ultrasound and the ABVS [26]. Five radiologists reviewed the volume data and detected between 74% and 88% of the lesions. Substantial agreement was found for BI-RADS® final assessment category (κ = 0.63).

Recently, Golatta et al. published data on 84 single breast examinations in 42 women [27]. Six breast diagnostic specialists interpreted the 3D-images. Based on the BI-RADS® classification the multiple kappa coefficient was κ = 0.35.

In our analysis, we found fair agreement between the two examiners, which correlates with the latter results (κ = 0.27). However, more data is needed to evaluate the performance of the ABVS in breast imaging convincingly.

Moreover, there are several reports comparing the ABVS with hand-held ultrasound [15,28-31]:

The first detailed description of the technical background and performance of the ABVS was published by our study group in 2011 [15]. In 2011, 50 ABVS datasets were evaluated by an independent examiner and accuracy, sensitivity and specificity were calculated as 66.0% (95% CI: 52.9-79.1), 100% (95% CI: 73.2-100) and 52.8% (95% CI: 35.7-69.2), respectively. Concerning these variables, our current study yielded comparable results (Table 5). Nevertheless, as both studies were conducted in the same institution, a comparison with the results from other study groups will be of greater interest.

In 2012, Lin et al. published data on 81 patients and compared ABVS to handheld ultrasound. The authors described a perfect sensitivity for both methods (ABVS: 100%, hand-held ultrasound: 100%), high specificity (95.0% and 85.0%, respectively) and, consequently, a high diagnostic accuracy (97.1% and 91.4%, respectively) [28]. This performance appears extraordinarily high. We suggest that further standardized studies in larger collectives should investigate if these expectations can be fulfilled.

In the same year, Wang HY et al. studied 239 lesions in 213 women who were scheduled to undergo biopsy. In this study, ABVS was similar to hand-held ultrasound in terms of sensitivity (ABVS: 95.3%, hand-held ultrasound: 90.6%), specificity (80.5% and 82.5%, respectively), and accuracy (85.8% and 85.3%, respectively) [29].

Recently, Wang ZL et al. published data on 153 patients with 165 breast lesions. The patients underwent mammography, ABVS and hand-held ultrasound. The authors reported no significant differences between ABVS and conventional ultrasound concerning sensitivity (ABVS: 96.1%, hand-held ultrasound: 93.2%), specificity (91.9% and 88.7%, respectively), and accuracy (94.5% and 91.5%, respectively) [30,31].

Compared to our data, there is a discrepancy mainly concerning the specificity, which was lower in our investigation (Table 8). Actually, we had a relevant number of healthy women without breast lesions (i.e. BI-RADS®-US 1) in our collective. This condition automatically increases the false-positive rate and actually reduces specificity.

**Table 8:** Data in the literature concerning sensitivity (SE) and specificity (SP) of the ABVS

**Author**	**N**	**SE**	**SP**
Wojcinski et al. 2011 [15]	50^a^	100%	52.8%
Lin et al. 2012 [28]	81^b^	100%	95.0%
Wang HY et al. 2012 [29]	239^c^	95.3%	80.5%
Wang ZL et al. 2012 [30]	165^c^	96.1%	91.9%
Current study, Examiner 1	100^a^	83.3%	68.3%
Current study, Examiner 2	100^a^	100%	51.2%

^a^volume data sets (including scans without lesions, BI-RADS®-US 1).

^b^patients.

^c^lesions.

Hence, the main difference of our study design in comparison to the above-mentioned studies is that we did not focus on the evaluation of known lesions alone, but also on the detection. Therefore, our examiners did not know whether there was a lesion in the particular volume or not. In the studies from the literature, the examiners were well aware, that there is definitely a lesion in the volume that requires biopsy (i.e. BI-RADS® 3 and above). This knowledge certainly has an influence on the evaluation of the lesion and the overall performance of the ABVS.

Our interpretation: the almost perfect performance of the ABVS as described in the literature is only valid for collectives of women with already pre-diagnosed breast lesions. In realistic collectives that also involve women without breast lesions (i.e. BI-RADS®-US 1) or with clearly benign breast lesions (i.e. BI-RADS®-US 2), our data may be more convincing.

Concerning the inter-observer agreement, Zhang et al. published data on 234 breast lesions from 208 patients who were examined with the ABVS [32]. Zhang et al. investigated the inter-observer agreement concerning the description of known breast masses according to the BI-RADS®-US lexicon. They found substantial agreement for lesion shape, orientation, margin, echo pattern, posterior acoustic features, calcification and final assessment, and fair agreement for retraction phenomenon and lesion boundary, respectively. Our study design did not focus the detailed description of lesions, but we showed fair agreement for the detection of breast masses. This aspect was not analyzed by Zhang et al.

Furthermore, there are reports in the literature about the ABVS that concentrate on the optimal scanning technique [16], the accuracy of measuring the cancer extent [33] and the detection of lesions located behind the nipple [34].

### Limitations of our study and limitations of the ABVS

All previously mentioned studies have the essential limitation of unicentric design and small patient collectives. This limitation also applies to our current study. Future study concepts should include multicenter design and larger, well-defined patient populations as described elsewhere [15]. Furthermore, we compared only two examiners. Although the results demonstrated fair agreement, the concordance should be confirmed with more observers. Another limitation of our study is the selection of the study population and the resulting concentration on BI-RADS®-US 1, 2 and 5 lesions which causes a certain bias. Therefore, the proportion of cases to controls is not representative of the whole population and BI-RADS®-US 0, 3 and 4 lesions are missing in the study population. We attempted to overcome this limitation by conducting a model calculation. Although this approach is statistically correct, it must be considered that, due to the small sample size and the vague estimation of the prevalence, the results must be carefully interpreted.

Moreover, the technology of the ABVS has some limitations per se. As previously described in the literature, automated breast ultrasound is limited in women with macromastia and pronounced ptosis [15,16]. In addition, the ABVS is not capable of scanning the axillary region. Today, sentinel node biopsy is the standard therapy for women that were preoperatively staged with a negative nodal status, which requires ultrasound of the axilla [35]. Furthermore, lymph node alterations may be the first sign in mammographically and/or sonographically occult breast cancer or other malignant diseases [36,37]. Therefore, additional conventional ultrasound of the axilla would be necessary after a suspicious ABVS scan.

Furthermore, we presume that even with optimal scanning technique the peripheral areas of the breast parenchyma are not fully covered by the ABVS. Additionally, shadowing artifacts occur in the retroareolar region and to a certain extent in the remaining breast volume. Therefore, a certain proportion of breast parenchyma may be lost in the volume data. This may reduce the diagnostic potential in comparison to hand-held ultrasound.

### Future implications of the ABVS

Finally, we have to discuss the question of which role the ABVS could play in future breast cancer diagnosis and breast cancer screening. Conventional hand-held breast ultrasound has a commonly accepted role as a complementary method to mammography, by adding to the diagnostic accuracy. Despite the well-known advantages, conventional breast ultrasound is a time-consuming, examiner-dependent and therefore costly procedure. Furthermore, breast cancer screening with additional breast ultrasound in low-risk women has not been established, as data concerning an effect on the overall survival is missing. Nevertheless, it is evident that breast ultrasound is capable of detecting previously occult cancers in women who had already undergone mammography and clinical examination [8,9,11,12]. However, screening larger collectives with conventional ultrasound is plainly not feasible. With the ABVS, we have a tool that could principally overcome these problems, and the ABVS may make ultrasound available to a larger number of women. Image acquisition with the ABVS can be efficiently performed by a medical assistant. Moreover, the ABVS allows a delayed interpretation of the images at any time, as well as second-readings. On the other hand, it has to be taken into account that we still experience a high number of time-consuming second-look ultrasounds due to the low specificity. This problem may be solved through second-readings by an independent examiner. Our proposed rules are relatively unusual, as, in order to improve the specificity, disagreement between the examiners would be classified as “negative” and only agreement that a scan is suspicious would be classified as “positive”. Therefore, further studies should include these concepts. However, we are convinced that the ABVS is a feasible method that can be easily integrated into the workflow of any inpatient or outpatient department.

We can imagine the ABVS being applied in breast cancer screening programs in the future and that additional examinations are offered to women at risk. However, we still have no data focusing on certain subgroups of patients (e.g. high-risk versus low-risk, ACR 1–2 versus ACR 3–4, etc.).

A prospective randomized controlled trial could answer the open questions. This trial should include patients with normal mammograms, some of whom will receive additional ABVS. The primary outcome variables would be the detection rate of breast cancer, the tumor stage and the overall survival of the two groups. Presumably, results could only be obtained if the collectives were sufficiently large, which makes it likewise unrealistic that a corresponding trial will actually be conducted.

## Conclusions

In conclusion, the ABVS has demonstrated high sensitivity and a fair inter-observer concordance in the evaluation of breast lesions. However, we have to overcome the problem of the high number of false-positive results in healthy women. Therefore, further prospective validation studies in larger collectives are necessary to define standard procedures in image acquisition and volume interpretation. Nevertheless, the ABVS is a reliable, technically mature and potentially beneficial imaging method. Therefore, we propose that the ABVS can be used in routine diagnostic procedures as an adjunct to mammography and conventional ultrasound, as previously described.

## Competing interests

The authors declare that they have no competing interest.

## Authors’ contributions

SW and SG contributed to the conception and design of the study and SW performed the ultrasound examinations and data collection. SG and AF performed the independent evaluation of the ABVS data sets. SW contributed to the statistical analysis and the interpretation of the data. SW and PS contributed to the writing of the manuscript. FD and PH conducted final reviews of the manuscript and FD provided methodological advice. All authors read and approve the final manuscript.

## Pre-publication history

The pre-publication history for this paper can be accessed here:

http://www.biomedcentral.com/1471-2342/13/36/prepub
